# Double hit mouse model of Parkinson's disease

**DOI:** 10.18632/oncotarget.13460

**Published:** 2016-11-18

**Authors:** Tommaso Schirinzi, Giuseppina Martella, Antonio Pisani

**Affiliations:** Department of Systems Medicine, University of Roma Tor Vergata, Rome, Italy

**Keywords:** Parkinson's disease, synaptopathy

Parkinson's Disease (PD) is the second most common neurodegenerative disorder, whose pathological hallmarks are the loss of dopaminergic cells of Substantia Nigra pars compacta (SNpc) and the brain accumulation of Lewy bodies, abnormal aggregates of α-synuclein. Neurodegeneration in SNpc leads to a prominent dopaminergic deficiency in the basal ganglia, responsible for motor disturbances. However, at network level, a broader neurochemical impairment involving several neurotransmitter systems, accounts for a number of additional non-motor symptoms. Available therapies give symptomatic relief but do not stop the neurodegenerative process. In more than the 85% of cases PD is an idiopathic disease, with evidence supporting a combined involvement of genetic susceptibility and environmental modifiers, a condition referred to as “double hit theory” [1, 2]. Therefore, modelling such a multifactorial condition may provide an ideal tool to explore both disease pathogenesis and novel therapeutic approaches.

The discovery of several familial forms of monogenic PD allowed generation of animal models that contributed significantly to the understanding of the disease pathogenic mechanisms, giving the opportunity to design preventive or disease-modifying treatments. In their recent publication, Martella and colleagues presented an interesting “double hit” model, based on the evidence that a single heterozygous mutation in the *PINK1* (phosphatase and tensin homolog-induced putative kinase 1) gene is sufficient to generate a genetic vulnerability to PD. In fact, while homozygous or compound heterozygous mutations in *PINK1* are responsible for a clinically manifest PD, heterozygosity causes subclinical dopaminergic dysfunctions in the absence of an overt phenotype [3]. The authors observed that heterozygous *PINK1* knockout mice, preserving normal motor behavior and intact nigral morphology, exhibit subtle impairment in dopamine release and corticostriatal synaptic plasticity [4]. Compelling evidence indicates that the disruption of synaptic activity, also termed “synaptopathy”, is an early event in the course of PD, directly related to the dopaminergic deficiency along which also progresses. Indeed, striatal dopamine, deriving from SNpc, is critical for the physiological occurrence of synaptic plasticity, a biological phenomenon by which corticostriatal synapses bidirectionally change their strength (Long-term potentiation or LTP and Long-term depression or LTD). Modulation of LTD and LTP plays a fundamental role for a correct motor control, and accordingly, abnormal plasticity is observed in a number of experimental models of PD [5]. The authors report that in heterozygous *PINK1* knockout mice, the subtle plasticity impairment may be significantly worsened by the exposure to environmental stressors such as pesticides [6], that are well-established environmental toxins, capable of inducing nigral degeneration [1, 6]. Rotenone inhibits the mitochondrial Complex I of the electron transport chain, thus exerting a dose-dependent toxicity on cellular metabolism causing the progressive damage of the SNpc dopaminergic neurons. By treating heterozygous *PINK1* mice with very low doses of rotenone, not sufficient to cause any neurodegeneration, the authors observed that the subtle synaptopathy observed in vehicle-treated mice and consisting in a selective loss of LTP, was further deteriorated with the failure of bidirectional plasticity. In agreement with the oxidative nature of the stressors, the loss of bidirectional synaptic plasticity could be prevented in this model by chronic administration of antioxidants, as α-tocopherol or Vitamin E [6].

A number of relevant points emerge from this study. First, this model, by using the corticostriatal synaptopathy as readout of early dysfunction, demonstrates the existence of a reversible phase in the disease course. Secondly, the presented “double hit” model may indeed represent a useful tool to test disease-modifying treatments. Finally, these data lay the basis to outline novel strategies of primary prevention for PD. In fact, clarifying the mechanisms by which the “second hit” occurs should help identifying populations at risk of PD, eligible for neuroprotective strategies, that might include also changes in living habits [1].
